# Fabrication and tunable reinforcement of net-shaped aluminum matrix composite parts via 3D printing

**DOI:** 10.1038/s41598-023-43514-y

**Published:** 2023-09-28

**Authors:** M. L. Seleznev, J. D. Roy-Mayhew, J. L. Faust

**Affiliations:** Markforged Inc., Watertown, MA USA

**Keywords:** Materials science, Structural materials, Engineering, Mechanical engineering

## Abstract

Advanced materials, such as metal matrix composites (MMCs), are important for innovation, national security, and addressing climate change. MMCs are used in military, aerospace, and automotive applications because of their exceptional mechanical and thermal properties, however adoption has been slow due to costly and onerous manufacturing processes. A new process using fused filament fabrication 3D printing has been developed to make net shape MMCs without tooling or machining. The process involves printing an alumina preform and then using pressure-less infiltration with a molten aluminum alloy to form the composite. Arbitrary shapes can be formed in this process—a brake lever and a flange are demonstrated—and the properties can be tuned by varying the ceramic infill geometric pattern and ceramic loading. By using 35 vol% continuous fiber reinforcement over 800 MPa strength and 140 GPa modulus are achieved for the aluminum composite, 3.4 × and 2 × the matrix aluminum properties.

## Introduction

Advanced materials are of strategic importance at the global scale as they are seen as key enablers to innovation, national security, and addressing grand challenges such as climate change^1,2^. As Deloitte calls out in their Advanced Materials Systems framework, advanced materials break existing trade-offs between cost and performance and will be necessary for companies to incorporate into products in order to stay competitive^3^. Polymer matrix composites (PMCs) are advanced materials praised for their adoption in the market^2^. In these a polymer matrix, usually epoxy, is reinforced with carbon, glass or Kevlar™ fibers. The resulting composite is a lightweight, high strength, and high stiffness enabler for high performance aircraft, wind turbines, and sporting goods, amongst other applications. However, as Maine and Garsney highlight, this innovation was not unlocked until process innovations were developed making it amenable to manufacturers^4^. Similar to PMCs, metal matrix composites (MMCs) were first fabricated by Stuhrke over 60 years ago^5^. This paper described as pioneering modern MMCs in^6^, discusses a diffusion bonded composite made up of five layers of unalloyed aluminum reinforced with 12–15% boron filaments. Although, MMCs are used in military (e.g., armor and ammunition), aerospace (e.g., engine components, landing gear), and automotive (e.g., brake disks) applications^7–13^, the adoption has lagged far behind PMCs due to difficulty in manufacturing—specifically shaping MMC parts. Indeed, the world PMC market in 2022 was estimated at 18.6 billion dollars^14^, while MMC market was estimated at just 2% of that ($366 million)^15^.

In an MMC, the metal matrix is reinforced with continuous or discontinuous carbon or ceramic fibers and/or particles. Aluminum matrix composites (AMCs) are the most common type of MMC and exhibit attractive properties that have not been obtained by PMCs. For example, due to the metal matrix, AMCs are excellent heat conductors and can be used at much higher temperatures while, due to the reinforcement, they exhibit much lower thermal expansion, have superior stiffness and strength at room and elevated temperatures and exhibit excellent wear resistance compared to unreinforced aluminum. However, ceramic reinforced AMCs are very challenging to machine using conventional methods and the difficulty grows sharply as reinforcement volume fraction increases^16^. Due to this challenge, only net-shape or near-net-shape manufacturing technologies, such as powder metallurgy, squeeze casting, or pressure infiltration casting have been viable manufacturing options for highly reinforced composites. However, these technologies require expensive equipment and tooling specific to the manufactured part. A promising technology to manufacture highly reinforced MMCs is pressureless infiltration, where self-supporting ceramic particulate preform is placed in a refractory reservoir filled with molten aluminum-magnesium alloy in a nitrogen atmosphere furnace^17,18^. In this approach, a molten alloy wicks into ceramic preform due to capillary forces and upon alloy solidification an AMC part is produced. Even though molds are not necessary for infiltration, this technology still requires a set of tooling for molding or pressing of particulate reinforcement preform, making it prohibitively expensive for many applications.

In recent years, additive manufacturing (AM), commonly known as 3D printing, has emerged as a versatile technology for fabricating plastic and metal components, eliminating the need for traditional manufacturing tools. This innovative process involves converting a computer-aided design (CAD) model into a physical object through a layer-by-layer building approach. Latterly, Dadkhah et al.^19^, Mostafaei et al.^20^ and Fereiduni and Elbesawi^21^ have conducted extensive reviews of the application of AM technology for manufacturing MMCs.

Most AM applications to MMCs fabrication involve laser and, occasionally, electron beam induced powder bed fusion techniques^19,21^, with much fewer instances of binder jet AM technology being employed^20^. The primary focus has been on aluminum and titanium as matrix materials, reinforced with diverse ceramic particles^19,20^. Additionally, copper, nickel, steel and tungsten MMCs have been produced using AM^20,21^. While powder bed fusion AM of MMCs has shown its fundamental feasibility, significant challenges arise due to inherent differences in properties, such as melting points and coefficients of thermal expansion, between the matrix and reinforcement. These disparities often lead to the occurrence of defects such as cracks and undesired reactions. Moreover, the high costs associated with manufacturing and equipment pose significant economic barriers, hindering the successful market penetration of this technology^19^. On the other hand, binder jet AM of MMCs frequently results in excessive porosity, with manufactured parts reaching levels as high as 50%^20^.

Hence, the primary objective of this study is to develop an additive manufacturing (AM) process capable of producing net shape Aluminum Matrix Composites (AMCs) with tunable properties, incorporating continuous reinforcement through fused filament fabrication (FFF) 3D printing. This approach offers exceptional flexibility in design while significantly reducing costs compared to traditional methods or other AM technologies for fabricating net-shape highly reinforced AMC parts.

**Figure 1:**
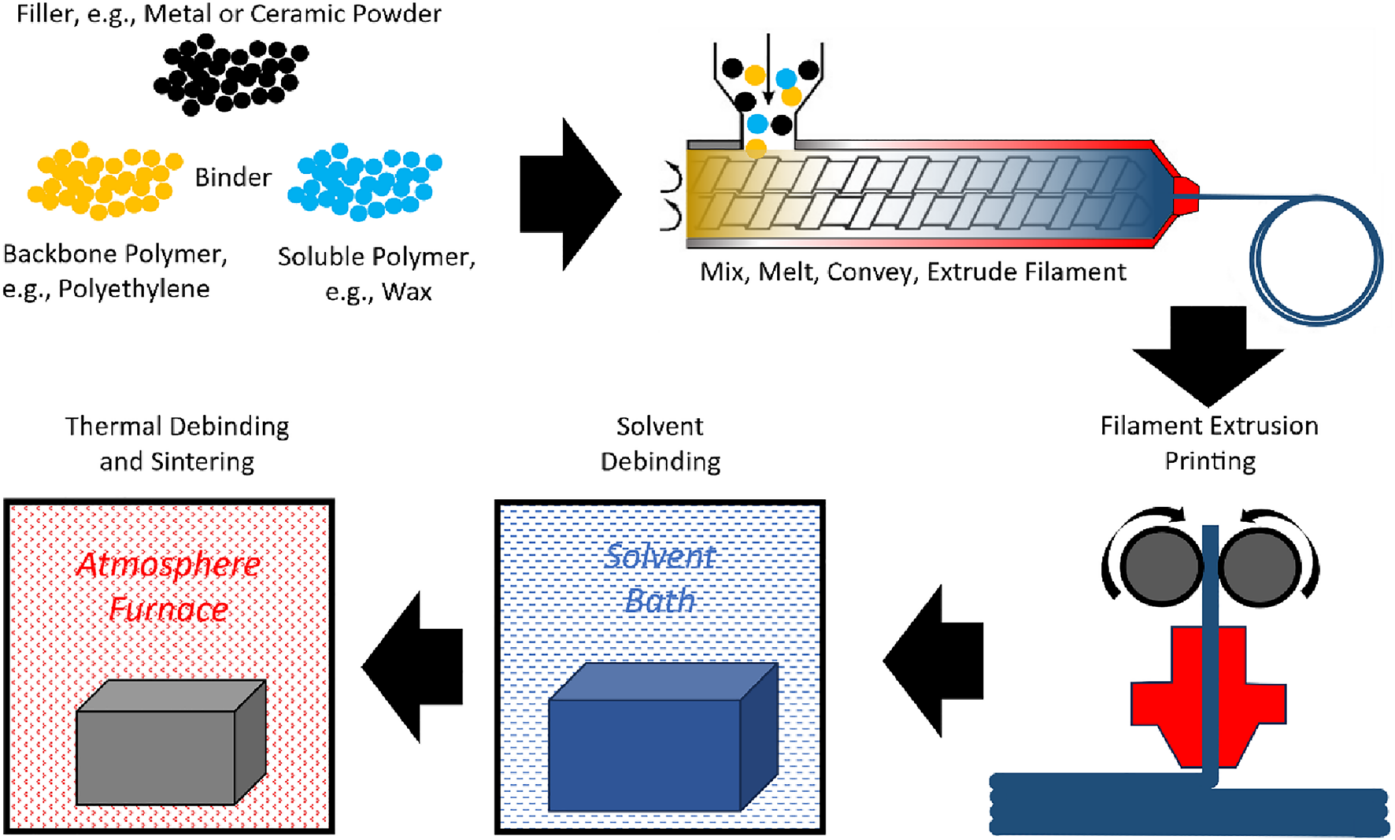
Metal or ceramic FFF process workflow description. Metal or ceramic powder filler is mixed with a binder that consists of a backbone and soluble polymers, and molten mix is extruded into a continuous filament, which is then used for filament extrusion 3D printing of a part. The printed part then undergoes solvent debinding, which dissolves out soluble polymer and opens interconnected microporosity inside the part and followed up with thermal debinding to remove backbone polymer and sintering of metal powder to densify the part.

Fused Filament Fabrication (FFF), the most widely adopted 3D printing technique, involves heating a thermoplastic filament and extruding it as a molten material bead. Over 100 companies have commercialized FFF in polymer-based materials, and a subset of these offers metal solutions that involve post-processing steps like debinding and sintering to achieve dense metal parts^22^. Flowchart and description of such technology is presented in Fig. 1. Notably, Markforged is among the companies providing both metal and polymer FFF platforms. Their polymer platform can incorporate continuous fiber reinforcement, including carbon fiber, glass fiber, and Kevlar™. The inclusion of continuous fibers significantly enhances the strength and modulus of the resulting parts, greatly surpassing those made solely from plastic materials and allows to optimize composite part topology as demonstrated in^23,24^.

This research work combines metal and composite technologies to showcase the FFF 3D printing of particulate ceramic (alumina) preforms. These preforms are either fully filled or feature internal infill pattern geometry, as well as being reinforced with continuous alumina fibers. Subsequently, a moldless, pressureless infiltration process is employed using a molten aluminum alloy to attain net shape aluminum matrix composites. The mechanical properties of the resulting AMC materials are presented, followed by the characterization of their microstructure. Finally, the mechanism underlying the infiltration process is thoroughly discussed.

## Results

**Figure 2:**
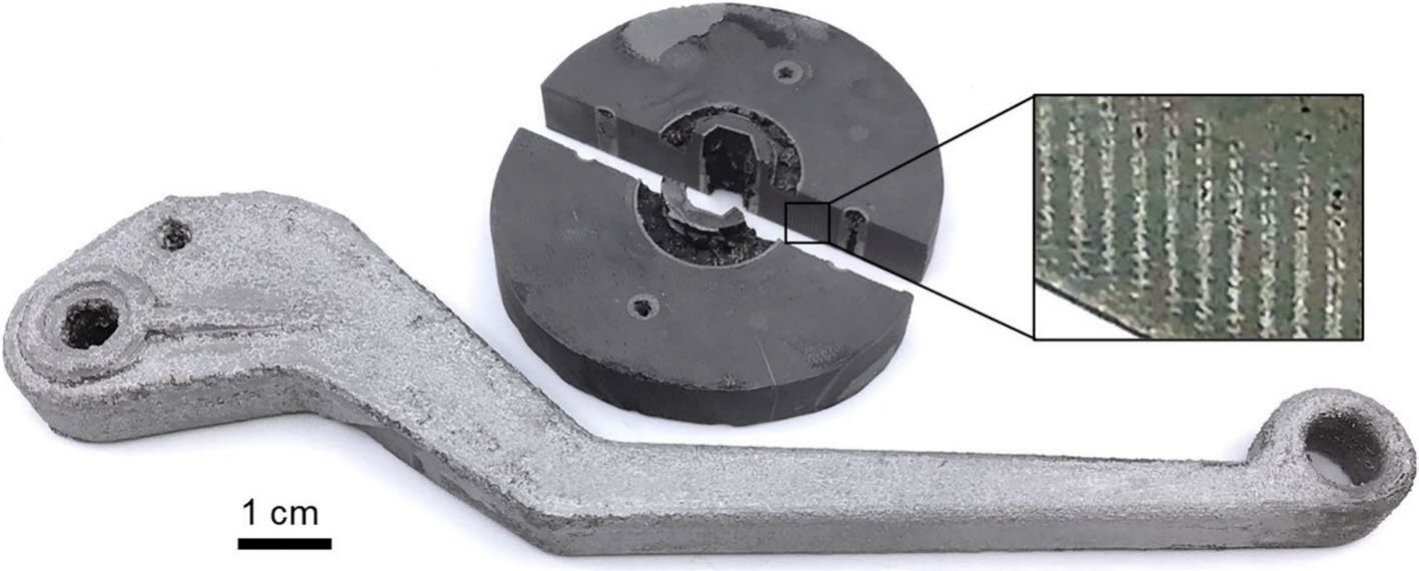
Demonstration AMC parts (break lever and flange) manufactured using novel 3D printing-based technology. The inset area of the flange shows infiltrated ceramic infill (dark areas) and surrounding aluminum matrix (light areas).

Using the methodologies outlined in the previous sections, we were able to fabricate both test samples, specifically 3-point-bend test beams, and characteristic parts that demonstrate the versatility of the process. Figure 2 showcases a flange and a brake lever, two three-dimensional shapes that would traditionally require custom tool sets for formation in conventional MMC processing.

In the cross-section image of the flange, the inset reveals two distinct sections. The shiny section corresponds to the matrix material, while the dark section represents the ceramic-reinforced portion. This section is formed by 3D printing of a ceramic component with internal infill, featuring a tunable pattern structure. It is worth noting that after the ceramic preform undergoes sintering and subsequent metal infiltration, a mesoscale structure composite material is produced. One can conceptualize the internal infill pattern as a macro-scale reinforcement structure encapsulated by the unreinforced aluminum matrix, with the pattern itself composed of microscopic ceramic particles embedded within the aluminum. For a comprehensive understanding of the fabrication process, please refer to the Methods section.

### Mechanical properties

**Figure 3:**
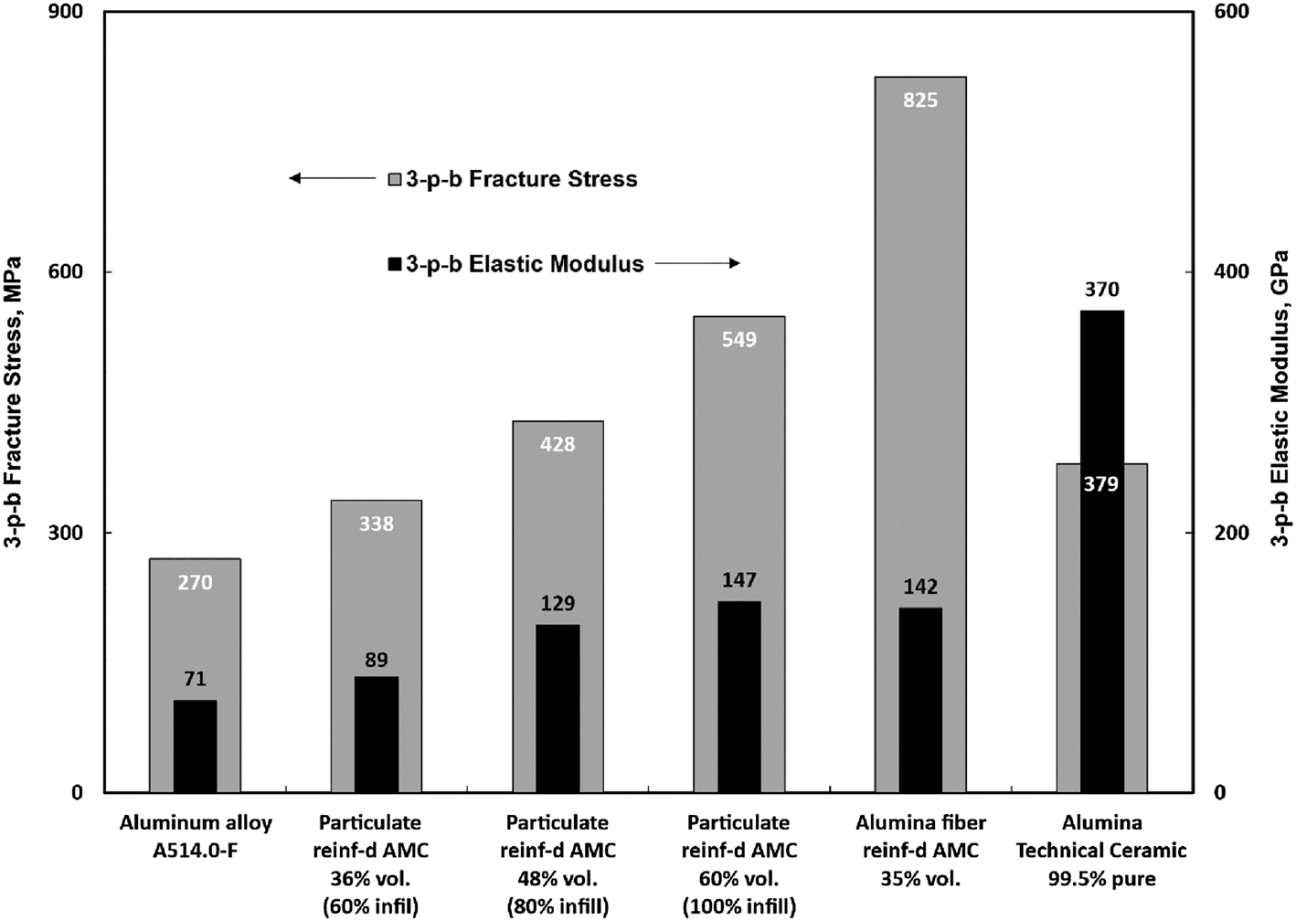
Strength and elastic modulus of particulate and continuous fiber reinforced AMCs tested in flexure versus particulate and fiber reinforcement volume fraction (infill density) of 3D printed preforms. Black bars are elastic modulus. Grey bars are strength. Aluminum and technical ceramic properties are adapted from literature^25,26^. A514.0-F aluminum casting alloy was used as a reference due to close composition, (4–5% magnesium based on matrix EDS composition analysis post infiltration).

Figure 3 illustrates the relationship between infill density and the fracture stress and elastic modulus of Aluminum Matrix Composites. Both fracture stress and elastic modulus show a nearly identical increase as the infill density rises, corresponding to an increase in the volume fraction of ceramic particulate reinforcement. In the explored interval, as the effective reinforcement volume fraction increases by a factor of 1.66 (from 36 to 60%), the AMC strength increases by 1.62 times (from 338 to 549 MPa) and the modulus increases by 1.65 times (from 89 to 147 GPa).

Comparing the fracture stress of particulate-reinforced AMC (100% infill) samples to unreinforced aluminum alloy^25^, the former exhibits more than a twofold increase. Furthermore, even with only 35% volume fraction of fiber reinforcement, the fracture stress exceeds the unreinforced alloy by more than threefold. When compared to an advanced technical ceramic^26^ known for its high strength and stiffness, the AMC surpasses the ceramic's strength at the 80% infill level and reaches 145% of the ceramic's strength with 100% infill. It should be noted that the elastic modulus of the ceramic is approximately twice as high as the highest reinforced particulate AMC or fiber-reinforced AMC. This outcome is expected since alumina serves as the reinforcement in composite, constituting only a portion of the material. Nevertheless, compared to the unreinforced aluminum alloy, the elastic modulus of the composite is 1.25–2 times higher, depending on the infill level.

**Figure 4:**
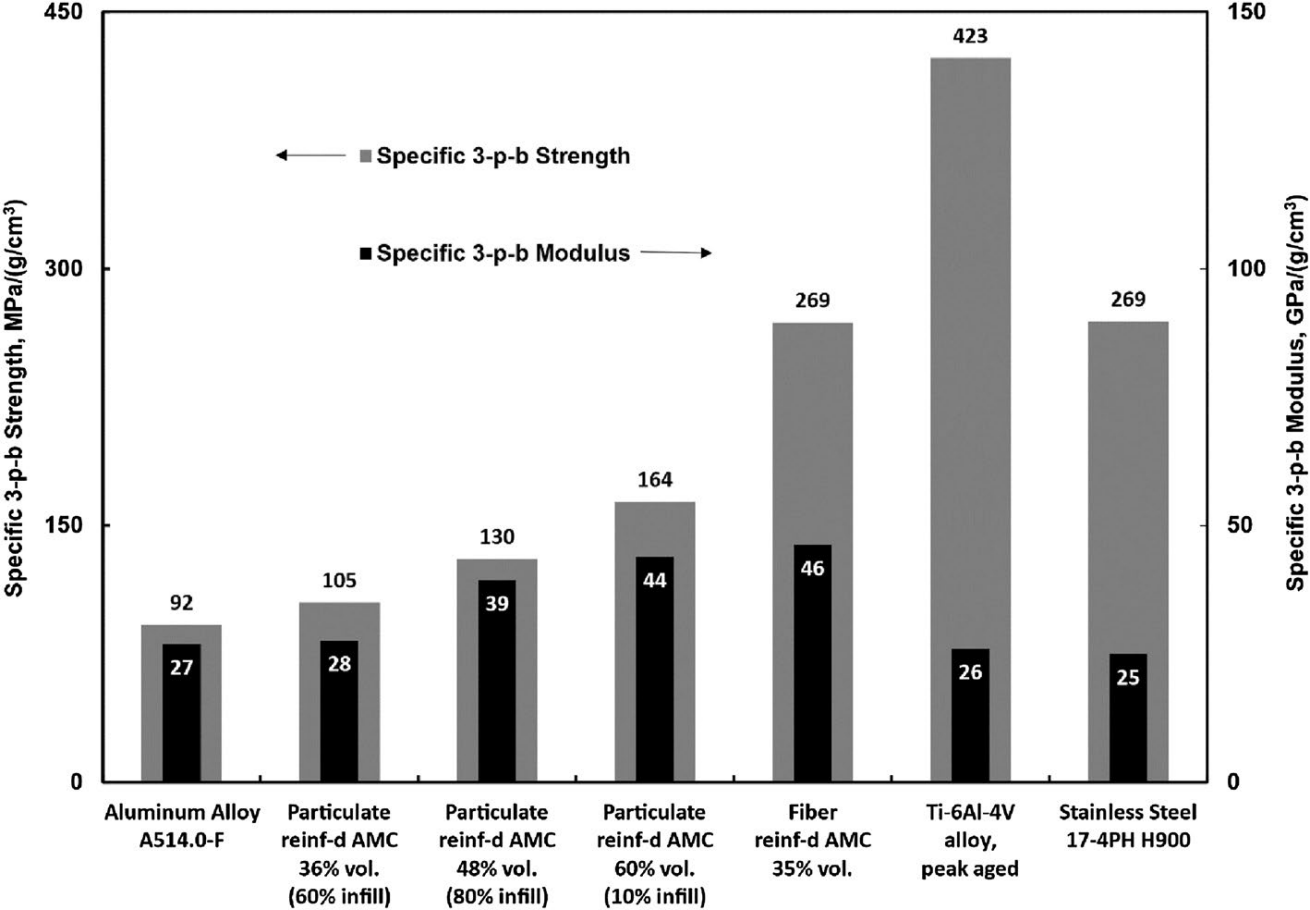
Specific strength and specific elastic modulus of particulate and continuous fiber reinforced AMCs tested in flexure versus particulate and fiber reinforcement volume fraction (infill density) of 3D printed preforms. Black bars are specific modulus. Gray bars are specific strength. Aluminum, Ti-6Al-V4, and stainless steel alloys properties are adapted from literature^25,27^.

AMCs are well-regarded for their superior performance compared to bulk alloys when considering specific properties normalized by density. To illustrate this, Fig. 4 presents a comparison of AMCs produced in this study alongside A514.0-F (an unreinforced aluminum alloy) and high-performance alloys such as peak-aged Ti–6Al-4V and wrought 17-4 PH stainless steel in the peak-aged H900 condition^25,27^.

The unreinforced alloys exhibit a narrow range of specific stiffness values, ranging from 25 to 27 GPa/(g/cm^3^). In contrast, the least stiff AMC with 60% infill density demonstrates a specific stiffness of 28 GPa/(g/cm^3^). Fiber-reinforced and 100% infill particulate-reinforced AMCs exhibit specific stiffness values of approximately 44 and 46 units, respectively, which are approximately 1.7 times higher than those of unreinforced metal alloys.

When considering specific strength, particulate-reinforced AMCs outperform unreinforced aluminum by a factor of 1.6 for 100% infill AMCs. However, they fall below the specific strength values of titanium and stainless steel alloys. Nevertheless, even at a 35% volume fraction of fiber reinforcement, fiber-reinforced AMCs match the specific strength of stainless steel.

From the perspective of an AMC part designer, a strategy to create a stiff and strong component would involve employing a hybrid-reinforced composite, where ceramic fibers are utilized in the most critical load-bearing regions.

**Figure 5:**
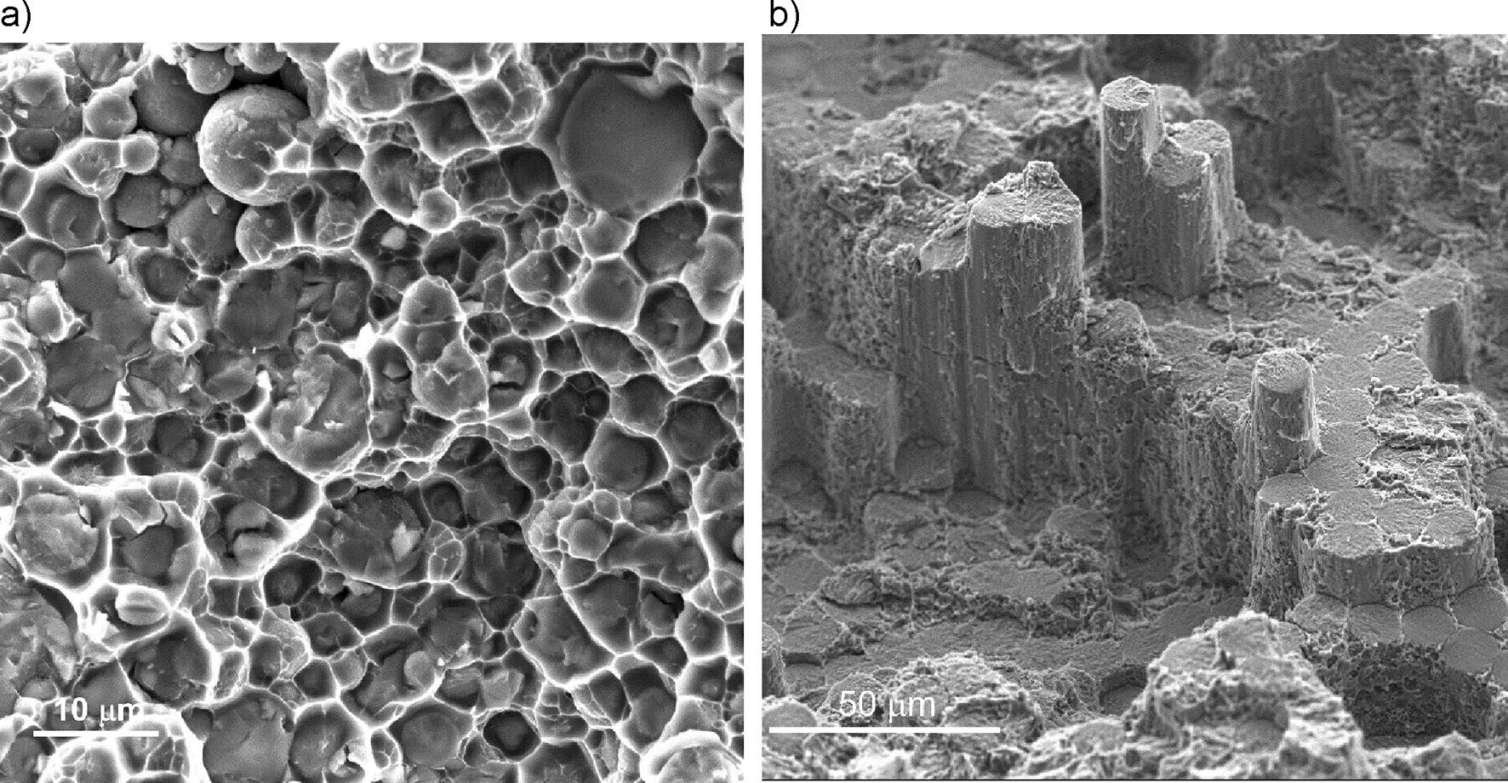
Fractographic images demonstrating strong adhesion of aluminum alloy matrix to alumina (**a**) particulate and (**b**) fiber.

The mechanical properties reported in the study can be compared with calculated values using widely accepted Voigt and Reuss models reviewed in^28^ that allow to estimate composite elastic modulus upper and lower bounds respectively. It should be noted that generally such models are applicable in case of strong adhesion between the reinforcement and matrix, which is supported in our case for both fiber and particulate composites based on SEM fractographic images—Fig. 5. Almost all the spherical reinforcement particles are cleaved in one plane during fracture. Similar cleavage is seen for fiber reinforcement, though some limited pull-out as a result of interface shear can be observed.

Assuming 36, 48 and 60% effective volume fraction of alumina reinforcement in aluminum matrix, the upper bound modulus estimates are 179, 215 and 250 GPa while the lower bound estimates are 100, 129 and 138 GPa, respectively. The measured values are 89, 129, 147 GPa, and as one may see are within approximately 10% of the lower bound Reuss model. This is understandable since the upper bound Voigt model is more suitable for a unidirectional continuous fiber reinforced composite case where elastic modulus is estimated using the rule-of-mixtures. Assuming 35% fiber volume fraction the model predicts 178 GPa modulus for fiber-reinforced composite, while the measured value is 142 GPa. The difference may be due to damage of continuous fiber during processing into a print filament and high temperature preform sintering operation.

**Figure 6:**
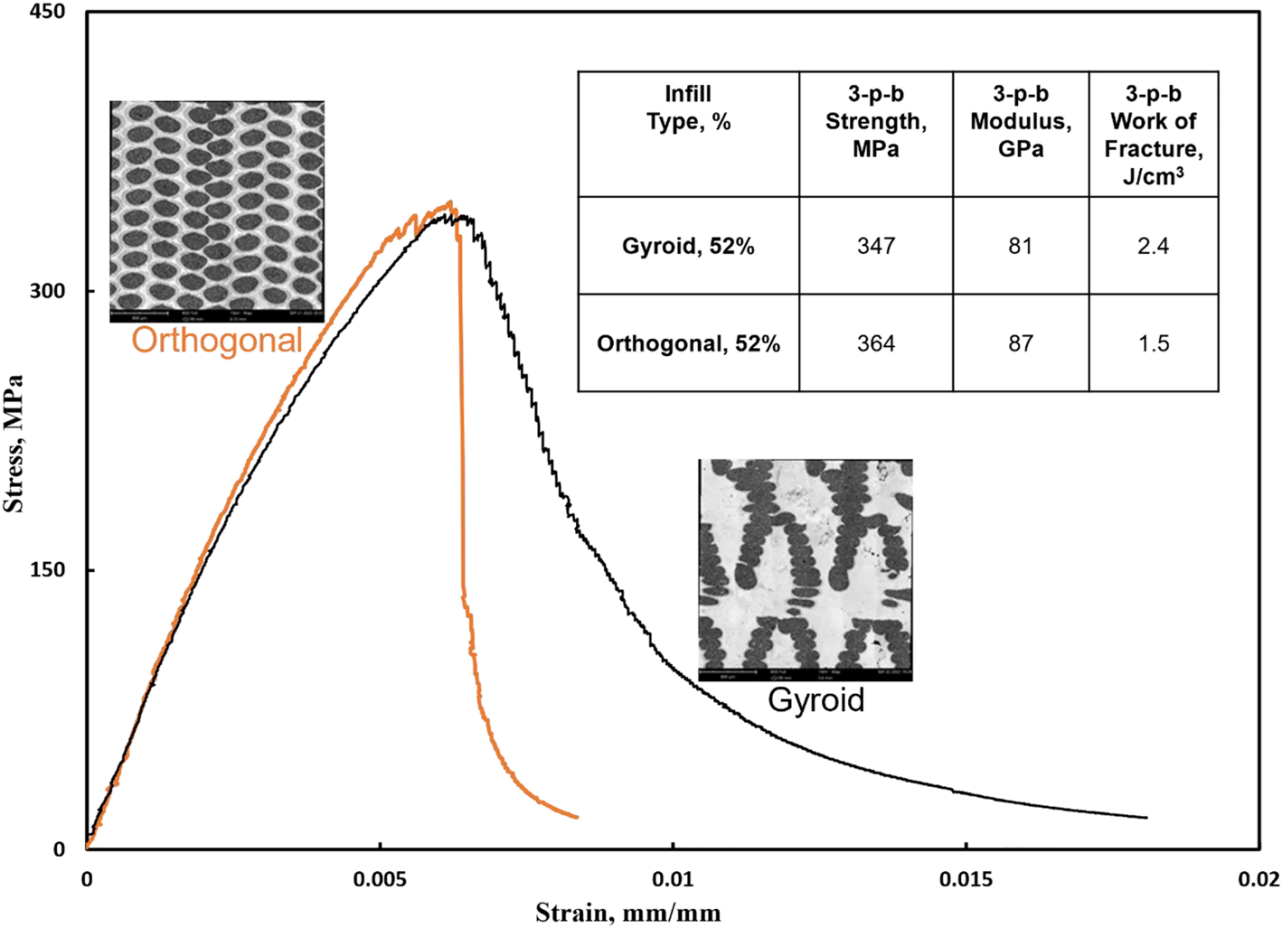
Typical 3-point-bend stress–strain curves for orthogonal (orange color) and gyroid (black color) infill pattern AMCs. In both cases infill density is the same at 52%. Mechanical properties are listed in the table and microstructures for both infill types are presented next to the curves.

It should be noted that all studied composites exhibited low nominal elongation prior to the fracture point of less than 1% due to high effective reinforcement volume fraction exceeding 36%, while within the printed beads ceramic volume fraction is even higher—60% by volume. However, in addition to varying the infill density, FFF 3D printing allows users to easily change geometric infill patterns when printing with ceramic particulate reinforced filament thus effecting printed composite toughness characterized by work of fracture. Two distinctly different infill geometric patterns, orthogonal and gyroid (seen in the inset of Fig. 6), were printed at 52% density—the maximum gyroid infill density that could be printed using the Metal X printer. As seen in Fig. 6, infill geometry had a negligible effect on strength and elastic modulus. However, infill geometry did have a strong effect on AMC composite toughness, with gyroid samples exhibiting × 1.6 greater work of fracture than the orthogonal patterned samples. While the gyroid infill sample fails gracefully, evidenced by the gradual stress decrease from maximum value while strain continues to grow, the orthogonal sample fails in a markedly more brittle manner. Such difference could be explained assuming that gyroid structure is more efficient at blunting propagating cracks, so more and more energy must be supplied to support an on-going fracture process.

### Microstructure

**Figure 7:**
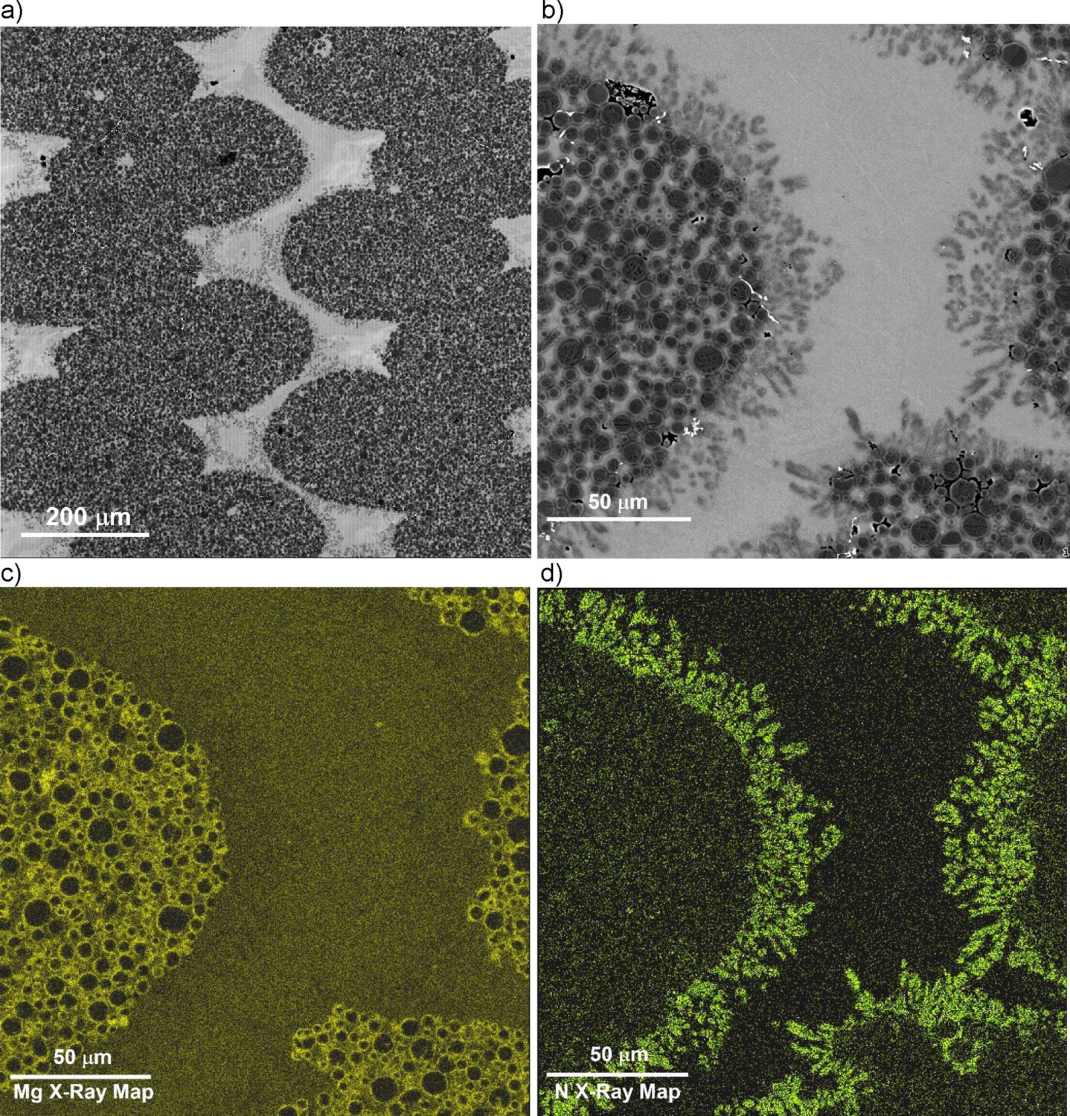
Typical microstructure of a particulate reinforced (80% infill) AMC manufactured using 3D printing technology. (**a**) BSE image; (**b**) high magnification view used for EDS; (**c**) Mg characteristic X-ray map; (**d**) N characteristic X-ray map.

**Figure 8:**
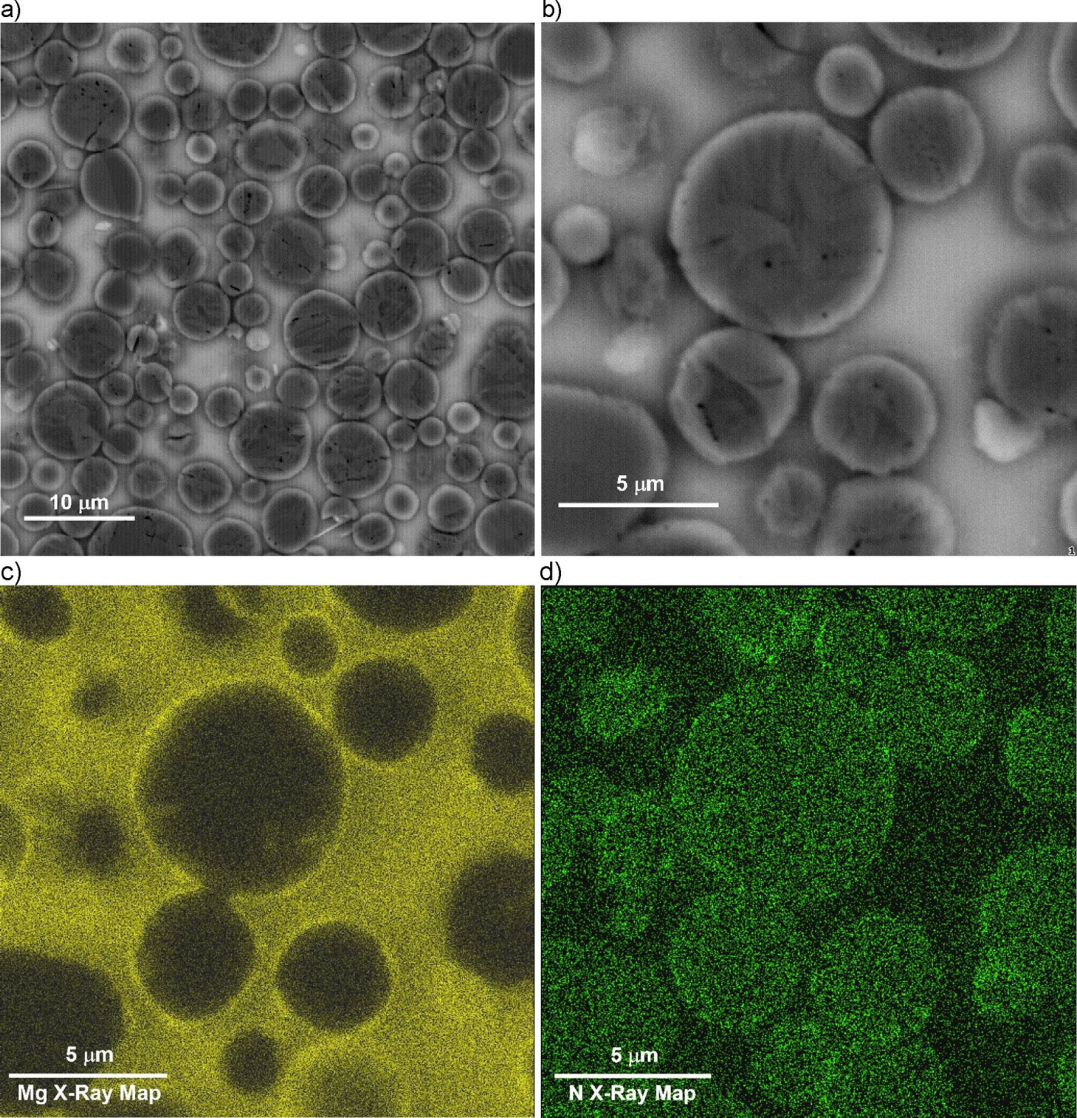
Microstructure within the infill region of an 80% infill density AMC sample. (**a**) BSE image; (**b**) high magnification region used for EDS; (**c**) Mg characteristic X-ray map; (**d**) N characteristic X-ray map.

Figure 7a depicts the typical microstructure of a particulate reinforced composite (80% orthogonal infill) featuring printed ceramic beads (dark grey) and an aluminum matrix (light grey). The ceramic beads are surrounded by lighter grey contrast particles, which are further illustrated in higher resolution in Fig. 7b. Detailed examination through characteristic X-ray maps (Figs. 7c,d) reveals that the peripheral grey particles, rich in nitrogen, lack oxygen and magnesium, indicating the presence of aluminum nitride. On the other hand, the interior of the printed ceramic bead presents a distinct difference. Figures 8a–d offer a closer look at this region. By analyzing the backscattered electron (BSE) images and conducting characteristic X-ray mapping of magnesium (Mg) and nitrogen (N) distributions, it is evident that aluminum nitride particles do not form rings around alumina particles and are generally absent within the beads. Instead, the surfaces of the alumina particles exhibit a significant magnesium presence, implying a reaction between alumina and the magnesium within the matrix alloy. Previous studies^29–31^ suggest that the resulting reaction product is a spinel known as MgAl_2_O_4_.

It is important to note that although the magnesium X-ray map in Fig. 7c may suggest that the interior areas of the ceramic beads have a higher magnesium content (brighter contrast) compared to the surrounding matrix, this is not the case. EDS analysis of the aluminum matrix composition in areas outside the infill beads and between alumina particles within the beads yields the same result of 4.5  ± 0.5% magnesium concentration. Therefore, the apparent difference in the magnesium X-ray map contrast between the beads and the surrounding matrix in Fig. 7c is due to the signal from the aluminum-magnesium spinel formed on the surface of the alumina particles. Additionally, Fig. 8c clearly demonstrates that the thickness of the MgAl_2_O_4_ spinel layer is significantly below 1 µm, despite the high concentration of magnesium in the infiltrant alloy. This observation aligns with the findings reported in^32^, which attribute the thinner spinel layer to a passivation effect resulting from high magnesium concentrations in the infiltrant aluminum alloy.

Another noteworthy observation (Fig. 7b) is a presence of a few “dry spots”—areas of incomplete infiltration in-between ceramic particles characterized by extremely dark contrast. The lack of infiltrant within a dry spot can be attributed to a variety of factors, including convoluted paths that hinder infiltrant penetration, metal shrinkage during solidification leading to voids, or the confinement of gases resulting in pore formation. It is important to note that all mechanical tests conducted in the study were carried out on materials reflecting their as-manufactured state, complete with these inherent defects. To mitigate the defects such process adjustments as infiltrant alloy composition changes, e.g., introduction of eutectic-forming silicon or post-processing steps, like hot isostatic pressing could be explored in future studies.

**Figure 9:**
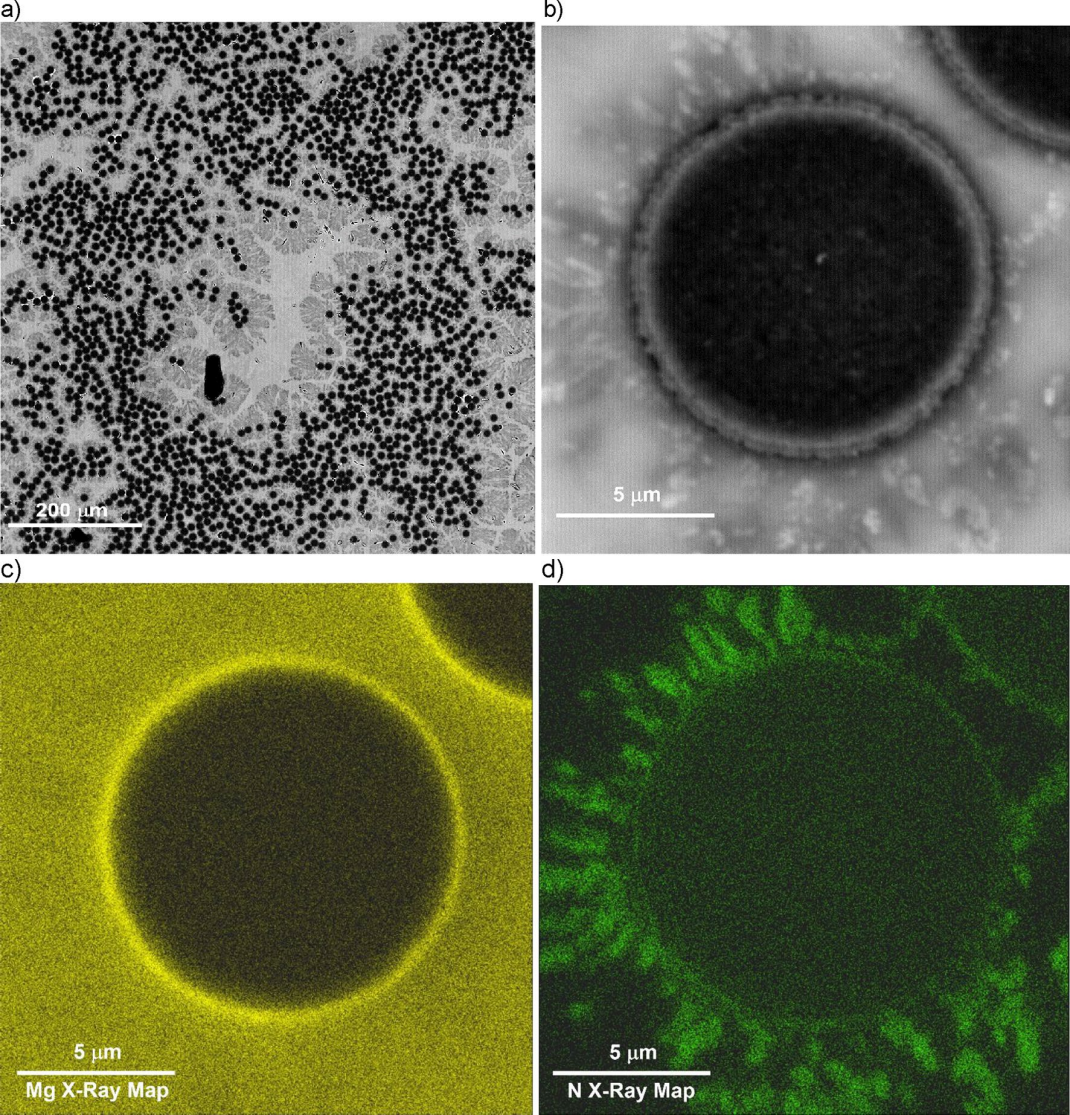
Microstructure of a fiber reinforced AMC manufactured using 3D printing technology. (**a**) Cross section in BSE contrast shown such that the fibers run perpendicular to the page. (**b**) higher magnification view used with EDS; (**c**) Mg characteristic X-ray map; (**d**) N characteristic X-ray map.

**Figure 10:**
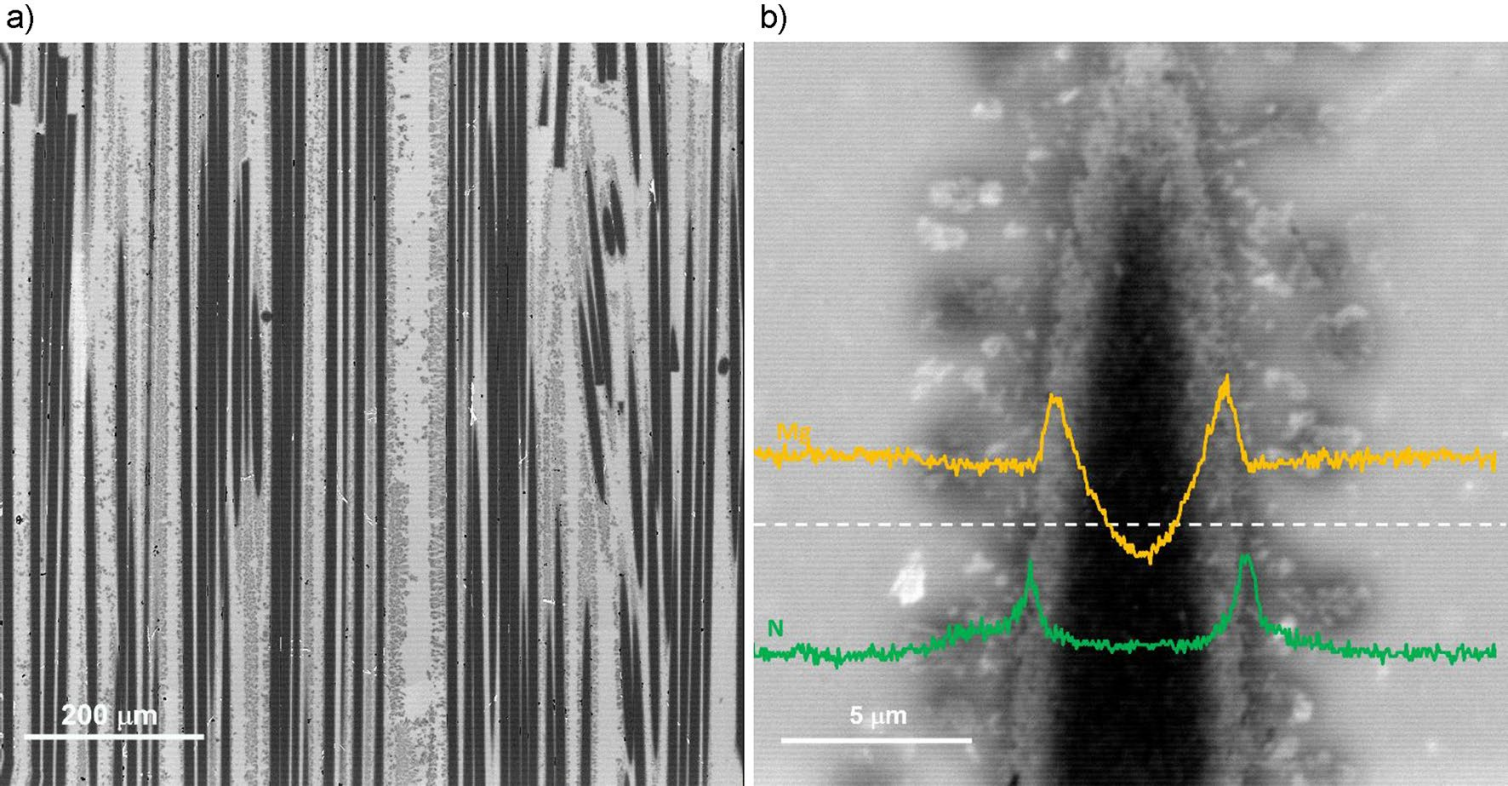
Microstructure of fiber reinforced AMC sectioned along fibers axis to better observe the fiber-matrix interface. (**a**) Low magnification BSE overview; (**b**) High magnification BSE micrograph with superimposed characteristic X-Ray line scans of Mg (top, orange) and N (bottom, green).

As depicted in Fig. 9a, the microstructure of the fiber-reinforced composite differs significantly from that of particulate-reinforced AMCs. While the spherical alumina particles are densely packed within the printed beads, the continuous fibers exhibit a loose arrangement at an overall volume fraction of 35%. In the fiber-reinforced AMCs, the micrograph reveals that aluminum nitride particles encompass not only fiber clusters but also individual fibers. The higher magnification image of an individual fiber in Fig. 9b highlights the presence of two reaction layers surrounding the fiber. Characteristic X-ray maps (Fig. 9c,d) demonstrate that one of the reaction layers is rich in magnesium, consistent with the MgAl_2_O_4_ spinel, while the other layer is rich in nitrogen, consistent with aluminum nitride (AlN), although the specific order of these layers is difficult to discern. To clarify the sequence of these layers, the microstructure of an alumina fiber-reinforced composite, polished in a plane parallel to the fiber axis, is examined (Fig. 10a). This technique allows for detailed imaging of the fiber-matrix interface, as any outer reaction layers are traversed by the polishing plane at a shallow angle and appear thicker in cross-section. A high-magnification backscattered electron (BSE) image, along with superimposed characteristic X-ray line scans, clearly reveals a layered structure of N–Mg–Fiber–Mg–N rich regions (Fig. 10b).

### Proposed infiltration mechanism

Microstructure observations presented in Section “Microstructure” shed light on the role of magnesium and nitrogen during the pressureless infiltration process of 3D printed ceramic preforms. Previous works^17,18,29,31^ proposed a mechanism for pressureless infiltration of pressed or injection-molded ceramic preforms, where the preforms were placed in a refractory container on top of an infiltrant magnesium-rich aluminum alloy stock within a nitrogen atmosphere furnace. This mechanism involves several steps: initially, the aluminum-magnesium alloy melts and contacts the surface of the ceramic preform. Subsequently, magnesium vapor released from the liquid alloy acts as an effective oxygen getter, reducing the aluminum oxide layer that coats the molten aluminum alloy surface thus facilitating flow. Concurrently, magnesium vapor reacts with nitrogen from the furnace atmosphere, forming magnesium nitride that coats the surfaces of ceramic particles within the preform. Finally, the aluminum-magnesium alloy wets the magnesium nitride coating, and under capillary forces, infiltrates the ceramic preform. During this process, the magnesium nitride coating on ceramic particles' surfaces is replaced by aluminum nitride.

In contrast, the microstructure observations in this study reveal a different sequence and mechanism for the pressureless infiltration process. First, the aluminum-magnesium alloy melts, and the liquid alloy contacts the bottom and internal walls of the 3D printed ceramic particulate reservoir. As the furnace temperature increases, magnesium begins to evaporate from the liquid alloy. The magnesium vapor acts as an effective oxygen getter, reducing the aluminum oxide layer that coats the molten aluminum alloy surface, thereby facilitating easier flow. As the furnace temperature exceeds 700 °C, the magnesium vapor reacts with the nitrogen in the furnace atmosphere to form magnesium nitride aerosol. This aerosol coats the printed reservoir, runners, ceramic preform surface, and the surface of the 3D printed ceramic particles that constitute outer layers of the preforms’ infill beads. The tightly packed 3D printed infill beads act as an absorption filter, preventing the penetration of the magnesium nitride aerosol into the bead’s interior. The aluminum alloy wets the surface of the 3D printed reservoir coated with magnesium nitride and begins to spread over the surface of the 3D-printed ceramic runners, also coated with magnesium nitride. This spreading is facilitated by a reaction wetting mechanism, where aluminum reacts with magnesium nitride, forming aluminum nitride. The spreading of the aluminum alloy can transport infiltrant alloy over lateral distances of up to 10 cm or more from the reservoir to the preform using the runners. As the aluminum alloy spreads over the preform surface, it begins to penetrate the interior of the preform, filling the areas between the printed beads that form the internal infill pattern. This penetration occurs because the outer surfaces of the beads are also coated with magnesium nitride. The aluminum-magnesium alloy forms relatively thick layers (hundreds of micrometers) as it spreads over the magnesium nitride coating, filling large voids between the 3D printed infill beads, even in preforms with low infill densities. After infiltrating the areas around the 3D printed preform infill, the aluminum alloy initiates the infiltration of the tightly packed spaces between the ceramic particles that make up the infill beads. This stage of infiltration also involves a reaction wetting mechanism, but it does not involve magnesium nitride. Instead, it takes place over shorter distances, comparable to half the width of an infill bead (~ 150 µm). In this stage, magnesium from the alloy reacts with alumina particles, forming a spinel layer on their surface. This mechanism is consistent with the absence of aluminum nitride inside the 3D printed ceramic beads and the presence of a micron-thick layer of magnesium–aluminum spinel on the surface of alumina particles.

The mechanism for fiber-reinforced preforms differs slightly. Since fiber-reinforced preforms are not tightly packed like 3D printed ceramic particle beads, the magnesium nitride aerosol can easily penetrate the preform and coat the surfaces of individual fibers. The liquid aluminum-magnesium alloy infiltrates the fiber preform due to excellent wetting with the magnesium nitride coating and subsequently reacts with the surface, forming aluminum nitride. In this mechanism, the aluminum nitride coating around the fibers does not act as a diffusion barrier for magnesium. Instead, magnesium reacts with the alumina fiber, forming a spinel layer on the fiber surface beneath the aluminum nitride coating.

In summary, there are significant differences between the legacy infiltration mechanisms and the proposed mechanism for the new 3D printing technology. First, unlike the mechanisms described in previous studies, the new infiltration process involves two distinct mechanisms. The first mechanism enables the transport of the infiltrant alloy over relatively long distances (up to 10 cm or more) and the filling of large open volumes (hundreds of microns) between infill beads or loosely packed fiber beds. This mechanism relies on the reaction wetting of the in-situ formed magnesium nitride by the alloy. The second mechanism facilitates the short-distance infiltration of tightly packed ceramic particle infill beads, relying on the reaction wetting of the alumina particle surfaces by the aluminum-magnesium alloy, resulting in the formation of a surface spinel layer.

Another significant difference is related to the infiltration process directionality. The legacy mechanisms assume an inside-out infiltration process, where the advancing infiltration front starts from the bottom of a part floating on a liquid infiltrant alloy pool and gradually spreads in-between ceramic particles inside the preform, reaching the surface last. However, the mechanism of the new technology suggests an outside-in process, where the liquid infiltrant initially spreads over the preform surface and then wicks into the preform volume.

These differences in the infiltration mechanisms highlight the unique characteristics and advantages of the new 3D printing technology compared to legacy methods. The ability to employ multiple infiltration mechanisms and the outside-in process offer enhanced control and flexibility in achieving desired infiltration outcomes.

## Discussion

Advanced applications demand high-performance materials and efficient fabrication processes to foster rapid innovation. The utilization of fused filament fabrication (FFF) 3D printing in conjunction with pressureless infiltration offers a viable means of producing net-shape MMC parts with unparalleled design flexibility, eliminating the need for specialized tooling. This approach empowers engineers to not only control the volume fraction of reinforcement but also shape the internal material architecture by determining the placement and pattern of ceramic infill. Consequently, a mesoscale structured composite material is achieved, allowing for tailored properties in different regions of a component. Moreover, the selective incorporation of continuous fiber reinforcement further enhances the ability to customize composite properties according to the specific requirements of a given part.

Our current research placed significant emphasis on investigating the mechanical properties of 3D printed composites, revealing that their behavior is predominantly shaped by the introduction of alumina particulate reinforcement. The effective volume fractions we have explored range from 36 to 60%. Within this range, we have observed a gradual increase in both strength and elastic modulus, exceeding those of unreinforced matrix aluminum alloy by a factor of two. Remarkably, continuous alumina fiber, a highly efficient reinforcement, achieved three times the strength of unreinforced alloy at only 35% volume fraction. Introducing particulate reinforcement at a 60% volume fraction increased the specific modulus of the composite by 1.6–1.7 times when compared to unreinforced aluminum, stainless steel, or titanium alloys. The same specific modulus improvement could be achieved with just 35% continuous fiber reinforcement.

Notably, even at the lower limit of the effective range, the volume fraction of reinforcement within the deposited beads remains high at 60%. As a result, all examined composites consistently exhibit a nominal elongation of less than 1% before reaching the point of fracture under flexural loading conditions. This limited elongation may seem constraining, but it aligns with the primary applications of highly reinforced composites, where stiffness is paramount. However, a significant trend emerges when we consider the work of fracture. Among composite materials with identical infill densities and effective reinforcement volume fractions, we observe noteworthy differences in this characteristic due to the tortuous paths crack propagation follows. Switching from an orthogonal to a gyroid infill pattern increases the work of fracture by a factor of 1.6. This observation underscores the superior damage tolerance of the gyroid meso-structure.

Our research also delves into the microstructure of 3D printed composites manufactured using this novel technology. We have proposed a new “outside-in” infiltration mechanism characterized by long-scale (infiltrant alloy spreading over part surface and filling the space between printed ceramic beads) and subsequent short-scale (infiltration of the printed beads) stages. Observations at metal-ceramic interface areas confirm the presence of a sub-micron spinel layer. This layer allows for effective load transfer without embrittling the interface—a crucial requirement for achieving robust and stiff metal matrix composite materials.

Considering these insights, we believe that this novel technology offers intriguing possibilities for exploring the meso-scale architecture of metal matrix composites. An especially promising avenue involves incorporating continuous fiber reinforcement alongside the existing three-dimensional lattice of discontinuous reinforcement. This synthesis of multifaceted architectures holds great potential for future experimental and theoretical investigations.

By making MMC production more accessible and providing comprehensive evidence of manufacturing concept feasibility, composite material properties, microstructure, and the underlying mechanisms of its’ formation, our aim is to empower manufacturers, expand the applications of this materials class and enable MMCs utilization in a wide range of industries.

## Methods

### Mechanical testing

Mechanical testing was performed on infiltrated samples that were ground to size using bonded diamond grinding disks and water as coolant and lubricant (120–600 grit in sequence). Flexural tests followed ASTM C1161-18 Standard Test Method for Flexural Strength of Advanced Ceramics at Ambient Temperature procedures using 6 × 8 × 90 mm rectangular cross section samples and 80 mm test span. All tests were performed using an in-house universal testing machine (Instron, model 3369) equipped with a 50 kN load cell. Strain data was collected using an Instron SVE 2 Non-Contacting Video Extensometer. Work of fracture was calculated as the area under the stress–strain curve using Microsoft Excel software. Unlike for ceramic, flexure tests are not commonly used for metals and alloys and their properties are reported tested in tension. Therefore, to make a comparison to AMC samples tested in flexure, ultimate tensile strength of A514.0-F alloy (170 MPa^25^) was multiplied by 1.6 to arrive at 270 MPa since for brittle materials flexure strength is about 1.6 times higher than tensile strength value^33^. Such difference arises because for equivalent sample cross sections, only bottom surface layers of a flexure test sample are subjected to maximum tensile stress compared to the whole cross section of a tensile test sample and so probability to encounter terminal defect is lower in flexure. Similar tensile to flexure conversion calculations were carried out for strengths of other commercial alloys referenced in the literature and used for comparison purposes in this study.

### Microstructure analysis

Metallography samples were cut from composite parts using a diamond disk saw (Isomet, Buehler), mounted in a conductive resin (Konductomet, Buehler), and mechanically ground and polished on an automatic grinder/polisher (E-4, Allied High Tech Products) using progressively finer SiC papers between 320 and 600 grit using water cooling and lubricating media, followed by 6-micron synthetic diamond suspension grinding on Chemomet cloth (Buehler) as a last step before polishing to remove traces of aluminum matrix oxidation. The final polishing step was performed using a Mastermet colloidal silica suspension on Chemomet cloth (both products of Buehler) for at least 18 min to ensure any grinding operations damage to soft aluminum matrix was removed.

Polished samples were then studied under SEM in backscattered electron mode (PhenomXL by Thermofisher). Fracture surfaces of mechanically tested samples were studied using secondary electrons mode. X-Ray spectroscopy was carried out using SEMs’ EDS spectrometer.

Density of the composite samples was evaluated using helium pycnometer (AccuPuc II 1340, Micrometrics).

### AMC manufacture—overall process steps sequence

**Figure 11:**
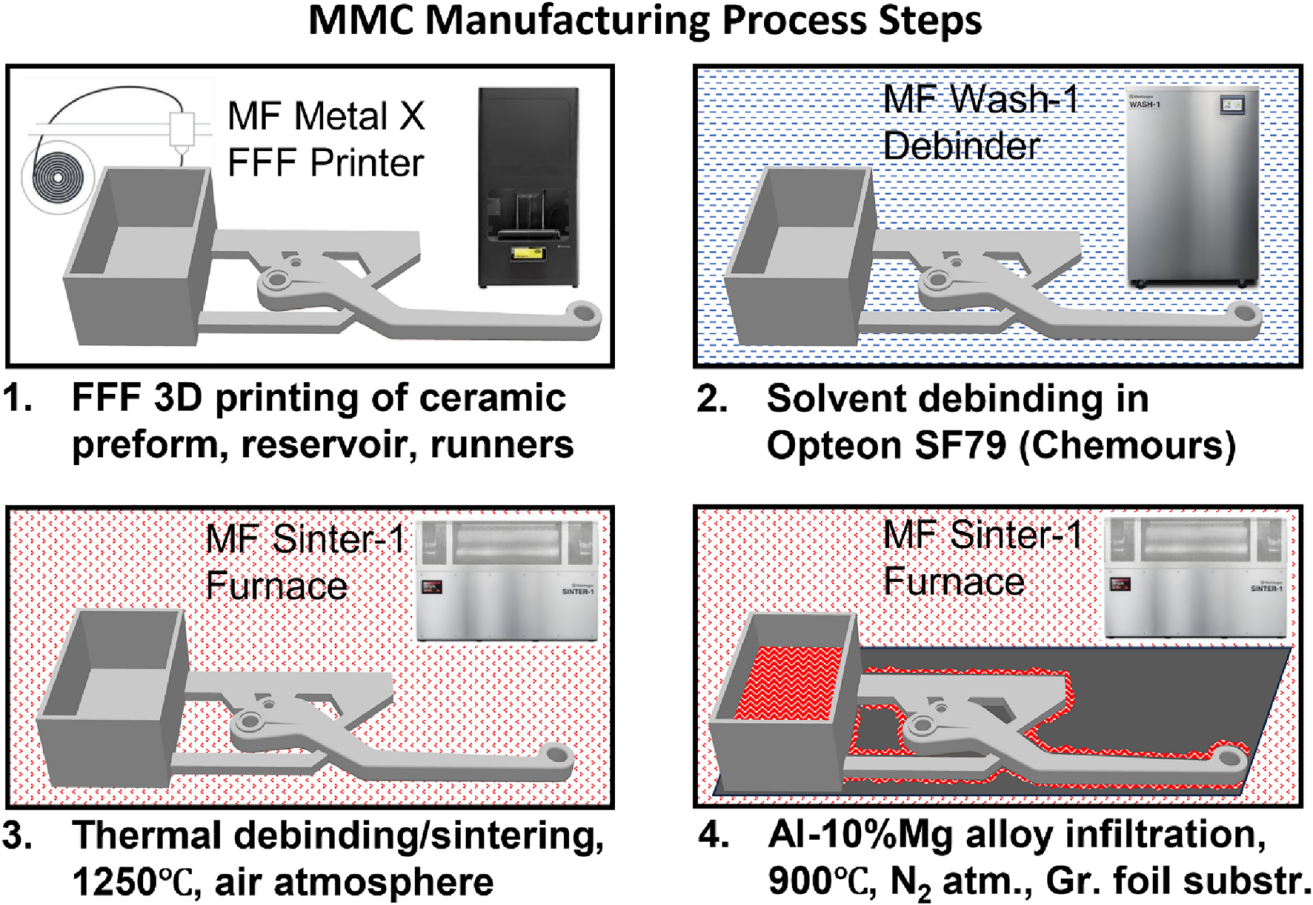
Novel 3D printing based MMC manufacturing process steps sequence.

Overall manufacturing process flow is shown in Fig. 11, while detailed description of operations is provided in the subsequent sections.

### AMC manufacture—preform fabrication

CAD files of each part were converted to .stl files and uploaded into the Eiger software (Markforged). Infill density (52%, 60%, 80%, 100%) and type (gyroid, rectangular) were selected in the software interface for each part. Considering that ceramic FFF filament was filled at 60% volume fraction, corresponding apparent ceramic volume fractions in AMC were 31%, 36%, 48% and 60%. Preform shape and internal infill structure changes grant tunability over the mechanical properties (stiffness, strength, fracture toughness) of AMC parts. Although not shown, other infill types (e.g., triangular, hexagonal) and infill densities (0–100%) could be selected.

**Figure 12:**
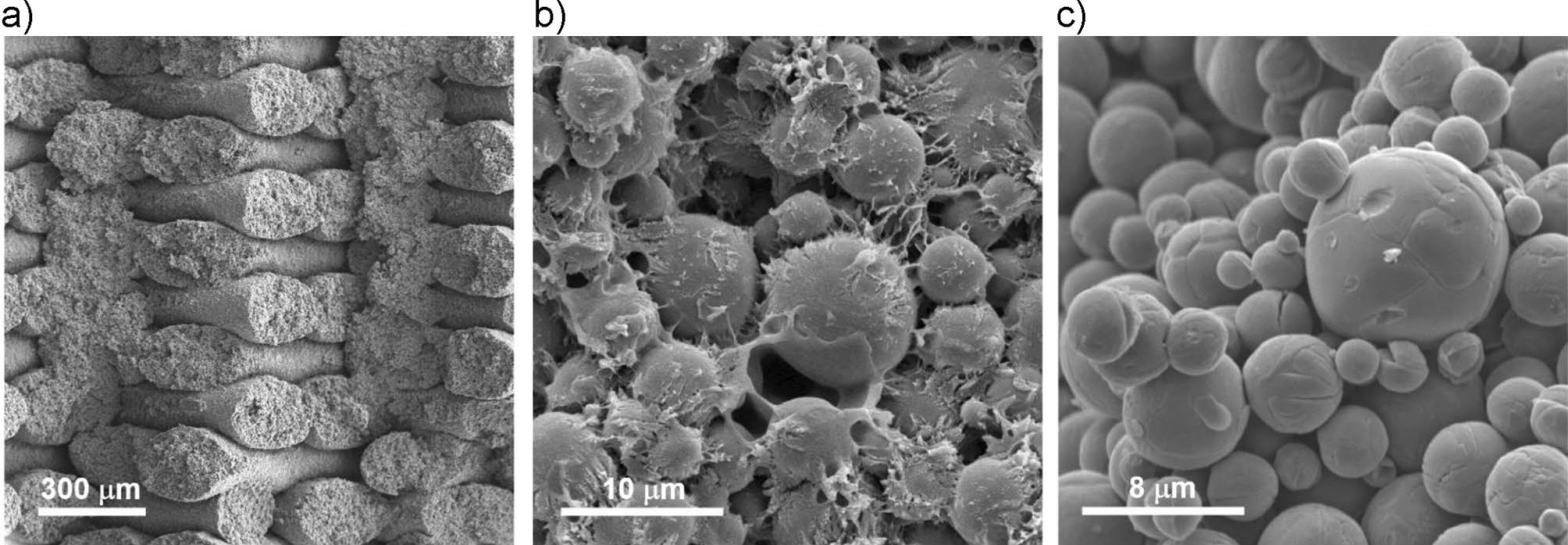
Macro and microstructure of a ceramic preform in BSE contrast after manufacturing operations of (**a**) printing; (**b**) solvent debinding; (**c**) thermal debinding and sintering.

Preforms were printed on a Markforged Metal X printer using a ceramic particle filled polymer filament. Ceramic particulate filled filament was produced by Markforged at 60% volume loading of spherical alumina particles d50 = 5 µm (Inframat Advanced Materials) using a proprietary binder system. Similar to metal and ceramic injection molding technology, the proprietary polymer binder consists of two parts—a solvent-washable component and a backbone polymer, which decomposes and burns off during a thermal debinding-sintering operation. Figure 12 shows macro and microstructure of a ceramic preform after printing, solvent debinding and thermal debinding using fractographic images of notched and cleaved samples. The printed preforms—see typical macrostructure depicted in Fig. 12a, were first washed in a solvent (Opteon SF79, Chemours) using commercial solvent debinder (Wash-1 Markforged). Microstructure of a preform after solvent debinding is presented in Fig. 12b, which shows ceramic particles bound by a spatial network of backbone polymer strands. The next thermal debinding and sintering operation was performed in air at 1250 °C for 3 h using a tube furnace (Sinter-1, Markforged). Oxidizing atmosphere burned off any of the backbone polymer residue cleanly to facilitate some ceramic particles, especially smaller ones, bonding at contact points—Fig. 12c.

By design, the size of ceramic particles used to print the preform was too large to result in any robust sintering at the 1250 °C temperature. To be successful AMC reinforcement ceramic particles must retain their shape and size and stay apart so they can be enveloped by aluminum matrix during infiltration. Therefore, a slight sintering of some alumina particles at contact points resulting in less than 3% overall (including debinding process contribution) linear dimensions shrinkage compared to as-printed preform was sufficient to result in a so-called bisque-sintered preform strong enough to be picked up by hand.

To demonstrate feasibility of AMCs with continuous reinforcement, stand-alone continuous alumina fiber reinforced preforms were printed using a Mark 2 composite printer (Markforged). The alumina continuous fiber filament was produced by Markforged using Nextel™-610 1k fiber (3 M) pre-pregged with a proprietary binder system, similar to that used in the ceramic particulate filament. Next, a particle-reinforced ceramic shell was printed using a Metal X printer, and the continuous fiber reinforced printed preform was placed into the shell during a pause in the shell printing process. After placement, the print was resumed. The hybrid preform was subjected to solvent debinding and then combined thermal debinding and sintering operations using the same equipment and regime as for particulate reinforced preforms. It is important to note that the shell cavity was printed oversized, so that the continuous fiber reinforced preform would have about 0.1 mm gaps on all sides when placed into the shell. Such gaps are necessary since the fiber reinforced preform consists of larger diameter (12 µm) fibers. As smaller particles facilitate sintering the particulate reinforced shell shrinks more than the fiber-reinforced preform core during sintering and could crack if no gap is provided. The outer particulate reinforced shell of composite was ground off after infiltration using diamond grit discs to result in a test sample reinforced with continuous fiber only.

### AMC manufacture—infiltration

The infiltration process developed in the present study was inspired by the so-called “PRIMEX” technique of pressure-less infiltration using an aluminum-magnesium alloy in a nitrogen atmosphere^17,18^. In its classical implementation a porous alumina or silicon carbide preform is placed into a refractory vessel filled with molten aluminum-magnesium alloy, such assembly sitting in a nitrogen atmosphere furnace, which alloy infiltrates the preform pores due to capillary forces. Magnesium plays an important role in the process as it evaporates from aluminum-magnesium alloy and coats ceramic particles of the preform with magnesium nitride after reacting with nitrogen atmosphere. Aluminum alloy then reactively wets magnesium nitride and forms aluminum nitride in its place, since at the infiltration temperature aluminum nitride is thermodynamically more stable than magnesium nitride^34^. Corresponding reactions can be written as follows:

$${\text{3Mg}} + {\text{N}}_{{2}} \to {\text{Mg}}_{{3}} {\text{N}}_{{2}}$$

$${\text{Mg}}_{{3}} {\text{N}}_{{2}} + {\text{2Al}} \to {\text{2AlN}} + {\text{3Mg}}$$

The infiltration is usually described as an inside-out process, where a molten alloy first enters the internal volume of a preform gradually spreading from within to the outside surface, which is coated with an infiltration barrier coating^18,35^. The most important difference of the present work is that the new technology utilizes FFF printed ceramic preforms that feature internal infill patterns. Preceding work emphasized that there is a limit on how large the gaps between ceramic particles inside a preform could be for an infiltration to be successful. For example, in^17^ the infiltration process failed when ceramic particles with 216 µm or higher average size were used. In the present work some printed preforms featured considerably larger internal openings (e.g., 400 + µm) between printed ceramic beads that defined the internal infill pattern, as well as openings between continuous fiber clusters. An additional difference is that the molten aluminum-magnesium alloy is not contained in a refractory container with the ceramic preform floating on top of it. In practice, it would be difficult, upon cooldown after infiltration, to separate infiltrated preform from a solidified pool of the excess infiltrant alloy necessary to insure complete infiltration. To avoid this problem a different set-up is used—pre-weighed amount of the infiltrant alloy stock (Al-10%Mg, Belmont Metals, NY) is put into rectangular-shaped reservoirs that are printed using the same ceramic as the preform. The reservoirs can be printed separately or as one with runners that are used to physically touch the preform and convey molten infiltrant alloy from reservoirs to the preform. Reservoirs, runners and preform could also be printed as one part if it is compact enough to handle without breaking. Reservoirs, preform and runners are then placed on a 0.5 mm thick graphite foil substrate (Amazon) folded into a tray shape, that is in turn supported by an alumina setter plate (Markforged) and the assembly is put into a tube furnace (Sinter-1, Markforged) with flowing ultrahigh purity (99.999%) nitrogen gas atmosphere (IGOs Welding Supply Co., Watertown, MA). Upon reaching 900 °C temperature the furnace is stabilized for 7 h and then cools down. Molten alloy first infiltrates the reservoir walls and floor and is then transported to the preform via runners that are in physical contact with both. It is important to note a crucial role in the process played by the graphite foil substrate. On one hand it helps to contain molten infiltrant alloy and not to allow it to spread far from under the ceramic parts. On the other hand, according to observations, the infiltrant alloy first actively spreads in the shallow gap between the sheet and ceramic parts on top of it, then over ceramic parts surface and only then starts to wick into ceramic parts interior. Whenever infiltration failed to proceed it was either because graphite foil substrate was not used or due to an insufficient amount of the infiltrant alloy being used. In the latter case the exterior of ceramic parts were infiltrated leaving interior dry. Therefore, unlike typical inside-out pressure-less ceramic infiltration processes described in the literature^17,18,35^, where infiltrant alloy gradually raises from the bottom of the preform through its’ bulk reaching outside surfaces last, in the new process the preform surface is infiltrated first and interior last. Another important observation is that colloidal graphite coating (Aquadag E, ABR Imagery) air-brushed onto a preform surface does not prevent the preform interior from being infiltrated but still helps to peel an excess surface aluminum skin layer from an infiltrated composite part.

## Data Availability

The datasets used and/or analyzed during the current study are available from the corresponding author on reasonable request.
